# Evodiamine Regulates Oxidative Stress and the JAK2/STAT3 Pathway to Modulate Apoptosis, Inflammation, Cell Cycle Arrest, and Migration in Periodontal Ligament Cells

**DOI:** 10.3390/antiox15040471

**Published:** 2026-04-10

**Authors:** Chuan Wang, Yuting Wen, Peiren Xu, Dong Yang

**Affiliations:** 1 State Key Laboratory of Oral & Maxillofacial Reconstruction and Regeneration, Key Laboratory of Oral Biomedicine Ministry of Education, Hubei Key Laboratory of Stomatology, School & Hospital of Stomatology, Wuhan University, Wuhan 430079, China; wangchuan0525@whu.edu.cn (C.W.); 2024283040033@whu.edu.cn (Y.W.); 2025283040032@whu.edu.cn (P.X.); 2 Department of Periodontology, School & Hospital of Stomatology, Wuhan University, Wuhan 430079, China

**Keywords:** evodiamine, antioxidant, anti-inflammatory, periodontitis

## Abstract

Periodontitis represents a primary etiological factor in tooth mobility, with oxidative stress contributing critically to periodontal tissue destruction. Evodiamine (EVO), a quinazolinocarboline alkaloid, exhibits multiple biological activities; however, its antioxidant effects and mechanism in periodontitis have not been elucidated. The aim of this study was to investigate the regulatory effect of EVO on oxidative stress in periodontitis and to explore the associated molecular mechanism. The results indicate that EVO exhibits potent antimicrobial activity against key periodontal pathogens and suppresses pathogen-induced ROS generation as well as the release of pro-inflammatory cytokines (IL-1β, IL-6, TNF-α) under periodontitis conditions. EVO binds specifically to the Kelch domain of KEAP1 with a strong binding energy (−11.67 kcal/mol), inhibits KEAP1–NRF2 interaction, and consequently upregulates the expression of antioxidant enzymes (HO-1, NQO1, GCLC, and SOD2), while downregulating the expression of iNOS, COX2, and NOX2. Furthermore, EVO inhibits the pro-apoptotic effect of the JAK2/STAT3 signaling axis and mitigates inflammation, alleviates cell cycle arrest, and promotes the migration and repair of periodontal ligament cells. Collectively, these findings suggest that EVO acts as a potential binder of KEAP1 that alleviates periodontal inflammation through modulation of oxidative stress and regulation of the JAK2/STAT3 pathway.

## 1. Introduction

Periodontitis ranks as the sixth most prevalent disease worldwide and is a leading cause of tooth loss in adults [1]. It is characterized by chronic inflammation of periodontal tissues resulting from bacterial infection [2]. The pathogenesis of periodontitis involves multiple factors, including bacterial infection, host immune response, and oxidative stress [3]. This persistent inflammation leads to the progressive and irreversible destruction of tooth-supporting structures, such as the periodontal ligament, gingiva, and alveolar bone [4].

Oxidative stress arises from a severe imbalance between the production of reactive oxygen species (ROS) and the antioxidant defense capacity of host tissues [5,6]. While these molecules are beneficial for cellular signaling and function under normal conditions, their excessive accumulation in the periodontal microenvironment becomes detrimental [7]. This imbalance induces oxidative stress, which serves as a major contributor to numerous diseases [8]. It directly damages cells, activates inflammatory pathways, and stimulates bone-resorbing osteoclasts, thereby exacerbating tissue injury [9].

Evodiamine (EVO) is a quinazolinocarboline alkaloid whose core structure consists of a fused heterocyclic system and additional modifications that enable interactions with various biological targets, including enzymes, DNA, and proteins. EVO is a bioactive component obtained from the nearly ripe fruits of *Tetradium ruticarpum* [10], and exhibits anti-inflammatory, anti-proliferative, and immunomodulatory activities [11]. EVO blocks phosphorylation of IκBα and NF-κB activation in zymosan-induced inflammation [12]. It upregulates SIRT1 and engages the PI3K/Akt pathway to suppress apoptosis and inflammation in intervertebral disk degeneration [13]. In bacteremia-induced inflammation, EVO regulates NLRP3 inflammasome activation and antibacterial responses by inducing α-tubulin acetylation [14]. Additionally, EVO ameliorates paclitaxel-induced neuropathic pain by inhibiting inflammation and maintaining mitochondrial antioxidant functions [15]. However, to date, the precise role of EVO in periodontitis, particularly its antioxidant mechanisms, remains entirely unexplored.

Kelch-like ECH-associated protein 1 (KEAP1) is a cytoplasmic repressor protein that senses oxidative and electrophilic stress [16]. It consists of five functional domains, with the BTB domain enabling homodimerization and the DGR/Kelch domain responsible for interacting with nuclear factor erythroid 2-related factor 2 (NRF2) [17]. NRF2 is a transcription factor belonging to the basic leucine zipper family, primarily regulating the transcription of genes encoding antioxidant enzymes [18]. Within the Neh2 domain of NRF2, two conserved motifs, DLG and ETGE, are present. The DLG motif targets the Neh2 domain for degradation. Under normal conditions, KEAP1 binds to NRF2 via a “hinge-and-latch” model [16], sequestering NRF2 in the cytoplasm and promoting its ubiquitination and degradation [19]. Oxidants or electrophiles can disrupt the KEAP1–NRF2 complex by covalently modifying cysteine residues in KEAP1, leading to NRF2 release.

Natural products are widely considered as candidates for periodontitis treatment. Paeoniflorin protects against periodontitis by suppressing the expression of MMPs, iNOS, and COX2 [20]. In experimental periodontitis, curcumin regulates the ROS response mediated by GPx, CAT, and SOD [21]. The KEAP1/NRF2 pathway is integrated into numerous cellular signaling and metabolic pathways, serving as a central regulatory node in oxidative stress. Studies focusing on the binding of phytochemicals to KEAP1/NRF2 have elucidated the regulatory mechanisms of this pathway with greater precision, while also demonstrating potent antioxidant activity. Curcumin alleviates murine inflammatory dermatitis by binding to Cys151 in the KEAP1 BTB domain, thereby modulating NRF2 transcriptional activity [22]. Covalent binding to the cysteine residues Cys77 in the BTB domain and Cys434 in the Kelch domain of KEAP1 is crucial for NRF2-mediated antioxidant regulation [23]. Natural products offer a comparatively safer regulatory effect. However, the specific mechanisms by which natural products, including EVO, regulate the KEAP1/NRF2 pathway still require further comprehensive investigation.

The aim of this study was to investigate the effect of EVO on the expression of pro-inflammatory cytokines in periodontitis condition, evaluate the changes in oxidative stress homeostasis, examine its effect on key periodontal pathogens, explore the molecular mechanism of its interaction with KEAP1, and further investigate its regulatory effect on apoptosis, cell cycle progression, and cell migration.

## 2. Materials and Methods

### 2.1. Primary Isolation and Culture of Cells

Premolars extracted for orthodontic reasons were collected as tissue sources. The procedure was approved by the Ethics Committee of the Hospital of Stomatology, Wuhan University (S0792502005), and informed consent was obtained from each patient. Periodontal ligament tissue was gently scraped from the middle third of the root surface and subsequently digested using type I collagenase together with 2.5% Dispase II (Roche, Grenzach, Germany). The isolated periodontal ligament cells (PDLCs) were then seeded in Dulbecco’s modified Eagle’s medium (DMEM, Cellmax, Beijing, China) supplemented with 10% fetal bovine serum (FBS, CellMax, China) and maintained at 37 °C in a humidified 5% CO_2_ atmosphere. Cells from passages 3 to 5 were used for the subsequent experiments.

### 2.2. Cell Viability and Survival Assays

Cell viability was assessed using the MTT assay. PDLCs were seeded in 96-well plates and cultured for 24 h. Cells were then treated with a concentration gradient of EVO (0, 0.1, 0.2, 0.5, 1.0, 5, 10 μM) for 48 h, followed by incubation with 10 μL MTT (M0280, Solarbio, Beijing, China) for 2 h. After removing the supernatant, 100 μL dimethyl sulfoxide was added to each well. Absorbance was measured at 490 nm using a microplate reader (Model 680, Bio-Rad, Hercules, CA, USA). Cell survival rate was calculated as a percentage relative to the untreated control.

The CellTiter-Glo^®^ Luminescent Cell Viability Assay kit (G9241, Promega, Madison, WI, USA) was used to quantify ATP levels for determining cellular metabolic activity, following the manufacturer’s protocol. The luminescent signal is proportional to the number of metabolically active cells.

### 2.3. Scanning Electron Microscopy (SEM)

Cells were seeded onto sterile glass coverslips placed in 24-well culture plates. After fixation with 2.5% glutaraldehyde (pH 7.4) at 4 °C, the samples were post-fixed in 1% osmium tetroxide. The fixed cells were dehydrated through an ethanol gradient. Critical-point drying with liquid CO_2_ was then performed, followed by gold–palladium sputter-coating. The samples were observed under a scanning electron microscope (SU8100, Hitachi, Hercules, Japan) at an accelerating voltage of 20 kV, and representative images were captured at 1200× magnification.

### 2.4. Measurement of Intracellular ROS Formation

Intracellular ROS levels in PDLCs were measured using the fluorescent probe DCFH-DA (D6883, Sigma-Aldrich, St. Louis, MO, USA). Cells were seeded into 96-well black plates at an appropriate density and subjected to the following treatments: (1) EVO alone (0, 0.1, 0.5 μM) for 3 h; (2) infection with *Porphyromonas gingivalis* (ATCC 33277), *Aggregatibacter actinomycetemcomitans* (ATCC 700685), or *Treponema denticola* (ATCC 35404); or (3) pretreatment with EVO for 3 h followed by co-culture with each pathogen for an additional 3 h. After treatment, the medium was removed, and cells were incubated with 10 μM DCFH-DA in serum-free medium for 30 min at 37 °C in the dark. Following two washes with PBS, fluorescence intensity was recorded at Ex/Em 488/525 nm using a SpectraMax i3x microplate reader (MiniMax 300, Molecular Devices, San Jose, CA, USA). Untreated cells served as the blank control, and ROS levels were expressed as relative fluorescence units and converted to percentages. All experiments were independently repeated three times, with six replicates per condition.

### 2.5. Western Blot Analysis

PDLCs were lysed using ice-cold RIPA buffer (cat. R0010, Solarbio, China). Protein samples were separated by SDS-PAGE and transferred onto PVDF membranes. After blocking with 5% non-fat milk for 1.5 h, membranes were incubated overnight at 4 °C with specific primary antibodies using GAPDH as the loading control. Primary antibodies included those against BAX, Bcl-2, Caspase-3, RAC1, CDC42, vimentin, E-cadherin, α-SMA, IL-1β, TNF-α, IL-6, iNOS, COX2, NOX2, HO-1, NQO1, GCLC, and SOD2 (detailed information is listed in the Supplementary File Table S1). Membranes were then incubated with appropriate secondary antibodies for 2 h, and protein bands were visualized using enhanced chemiluminescence. Band optical density (OD) was quantified using Quantity One v4.2 software (Bio-Rad, USA), and protein expression levels were calculated based on OD values.

### 2.6. Quantitative Real-Time Reverse Transcription Polymerase Chain Reaction (qRT-PCR)

Total RNA was extracted from cells cultured under various conditions for 48 h using TRIzol reagent (cat. 15596026, Thermo Fisher, Waltham, MA, USA). The mRNA expression levels of p21, p53, Cyclin E1, Cyclin B1, CDK1, and CDK2 were assessed by SYBR Green-based qRT-PCR on an iCycler Real-Time PCR system (iQ5, Bio-Rad, USA), with GAPDH serving as the reference gene (detailed information is listed in the Supplementary File Table S2). Primer sequences were designed using Primer Express v3 software (Applied Biosystems, Foster, CA, USA) based on GenBank entries and validated via Primer-BLAST v2.5 (NCBI, Bethesda, MD, USA). Relative changes in gene expression were calculated using the 2^−∆∆Ct^ method, and all experiments were performed with three independent replicates.

### 2.7. Atomic Force Microscopy (AFM) Imaging

The key periodontal pathogens (*P. gingivalis*, *A. actinomycetemcomitans*, and *T. denticola*) were cultured anaerobically. Mature biofilms formed after 48 h treatment with or without EVO were subjected to AFM imaging (DimensionIcon, Bruker, Billerica, MA, USA) in ScanAsyst mode at a 10 nm scale to determine their three-dimensional thickness and surface roughness. The thickness and coverage area of the biofilm were quantified using ImageJ software v1.54 (NIH, Bethesda, MD, USA). Bacterial growth curves were also plotted to evaluate the inhibitory effect of EVO on bacterial proliferation.

### 2.8. Flow Cytometric Analysis of Cell Cycle and Apoptosis

Flow cytometry (Calibur, BD, Franklin Lakes, NJ, USA) was used to assess the impact of EVO on cell cycle distribution and apoptosis. For cell cycle analysis, PDLCs were harvested after treatment for 24 h, washed with PBS, and then fixed overnight at 4 °C with pre-cooled 75% ethanol. Cells were centrifuged, resuspended in PBS, stained with 1 mg/mL RNase A at 37 °C for 30 min, and then with 50 μg/mL propidium iodide (PI) for 10 min. Apoptosis was quantified using the Annexin V-FITC/PI double staining kit (556547, BD, USA). PDLCs treated with various concentrations of EVO (for 24 h) were then collected, washed, and resuspended in 1× binding buffer. The cell suspensions were incubated in 5 μL Annexin V-FITC and 10 μL PI in the dark at room temperature for 15 min. Samples were analyzed immediately using a FACSCelesta flow cytometer (Calibur, BD, USA) and data were processed using FlowJo software (Version 10.8, TreeStar, Ashland, OR, USA) to categorize the cells as viable (Annexin V^−^/PI^−^), early apoptotic (Annexin V^+^/PI^−^), late apoptotic (Annexin V^+^/PI^+^), or necrotic (Annexin V^−^/PI^+^) cells.

### 2.9. Drug–Target Binding Stability Experiment

The DARTS assay was performed to identify the potential target of EVO in PDLCs. Total protein from PDLCs was extracted and incubated with 50 μM EVO or equivalent volume of DMSO (control) at 4 °C for 2 h. Samples were then treated with a gradient of protease concentrations (1:1500, 1:1200, 1:1000, 1:800) at room temperature for 30 min. Reactions were terminated by adding 5× SDS loading buffer, followed by separation on 10% SDS-PAGE gels. Subsequently, proteins were stained with Fast Silver Stain Kit (P0017S, Beyotime, Shanghai, China) and images were acquired using a ChemiDoc MP Imaging System (Bio-Rad, USA). Band densities were quantified using Image Lab v6.0.1 software (Bio-Rad, USA).

The drug–target binding stability validation assay was conducted under varying EVO concentrations and different temperatures. Similar to the DARTS assay, after treatment with protease at a 1:1000 ratio, different concentrations of EVO (0, 1, 0.5, 0.1, 0.05 μM) were added, and the binding stability between EVO and KEAP1 was monitored by Western blotting. To evaluate the binding stability through temperature-dependent KEAP1 degradation, seven temperature gradients were established (42, 45, 48, 51, 54, 57, and 60 °C). Each group was treated with and without 1 μM EVO, followed by Western blot analysis to assess the stability of the EVO–KEAP1 interaction.

### 2.10. Molecular Docking and Flexible Molecular Dynamics (MD) Simulations

To investigate the interaction between EVO and KEAP1 at the atomic level, a combined strategy of molecular docking and MD simulations was employed. The three-dimensional structure of the KEAP1 Kelch domain was obtained from the Protein Data Bank (PDB ID: 8a46). EVO (CAS: 518-17-2) was docked into its active pocket using AutoDock Vina 1.1.2 software (Scripps Research, La Jolla, CA, USA) to determine binding affinity and identify interaction sites, with all visualizations performed in PyMOL 2.5 (Schrödinger, New York, NY, USA). MD simulations were conducted using the AMBER20 package (UCSF, San Francisco, CA, USA). The KEAP1 structure was parameterized with the FF19SB force field, and EVO parameters were generated via the ANTECHAMBER module (AmberTools 20) using the GAFF2 force field. The system was solvated in a TIP3P water box and neutralized with Na^+^/Cl^−^ ions. After energy minimization and equilibration, three independent 100 ns production runs were performed under NPT conditions. Trajectory analyses focused on root-mean-square deviation (RMSD), root-mean-square fluctuation (RMSF), radius of gyration (Rg), and solvent-accessible surface area (SASA). The MM-PBSA method was employed to calculate the binding free energy, and energy contribution profiles were plotted. Using RMSD and Rg as reaction coordinates, a free energy landscape (FEL) was built based on principal component analysis to identify the conformational ensembles and the lowest energy state of the complex.

### 2.11. Cell Migration Assay

Cell migration was assessed using a scratch wound healing assay. PDLCs were seeded at an appropriate density in 12-well plates and cultured to full confluence. Uniform scratches were created using a sterile 10 μL pipette tip, and detached cells were washed away with PBS. The medium was replaced with serum-free DMEM containing different treatments: blank CTRL, PD condition, PD+EVO, PD+siNRF2+EVO, and PD+ovJAK2+EVO. After 36 h, cell nuclei were stained with DAPI, and images were captured using an inverted microscope (Eclipse Ts2, Nikon, Tokyo, Japan) under a 10× objective. The number of migrated cells was counted, and the migration percentage was calculated using ImageJ software.

### 2.12. Cell Immunofluorescence

To assess phenotypic changes, immunofluorescence staining of α-smooth muscle actin (α-SMA) was performed. PDLCs were divided into the following groups: PD condition, PD+EVO, PD+siNRF2+EVO, and PD+ovJAK2+EVO. After treatment, cells were fixed with 4% paraformaldehyde, permeabilized with 0.1% Triton X-100, and blocked with 5% BSA. Samples were then incubated overnight at 4 °C with a mouse anti-human α-SMA monoclonal primary antibody (1:200, Abcam, Waltham, MA, USA), followed by incubation with a Cy3-conjugated goat anti-mouse secondary antibody (1:400, Dianova, Hamburg, Germany) at room temperature in the dark for 1 h. Nuclei were counterstained with DAPI. Images were acquired using a fluorescence microscope (BX51, Olympus, Tokyo, Japan), and fluorescence intensity was quantified with ImageJ software. Experiments were independently repeated three times, with at least five random fields analyzed per sample.

### 2.13. Statistical Analysis

Continuous data are presented as mean ± standard error of the mean. One-way analysis of variance (ANOVA) was used for multi-group comparisons, followed by Tukey’s honestly significant difference (HSD) post hoc test for specific pairwise comparisons. Student’s *t*-test was employed for comparisons between two groups. Categorical outcomes are expressed as percentages and were compared using the chi-square test. All data were analyzed with SPSS 22.0 (IBM, Chicago, IL, USA) and visualized using GraphPad Prism 9 (GraphPad Software, San Diego, CA, USA) and Origin 2022b (OriginLab, Northampton, MA, USA). A *p*-value < 0.05 was considered statistically significant.

## 3. Results

### 3.1. Effect of EVO Treatment on PDLCs Status

**Figure 1:**
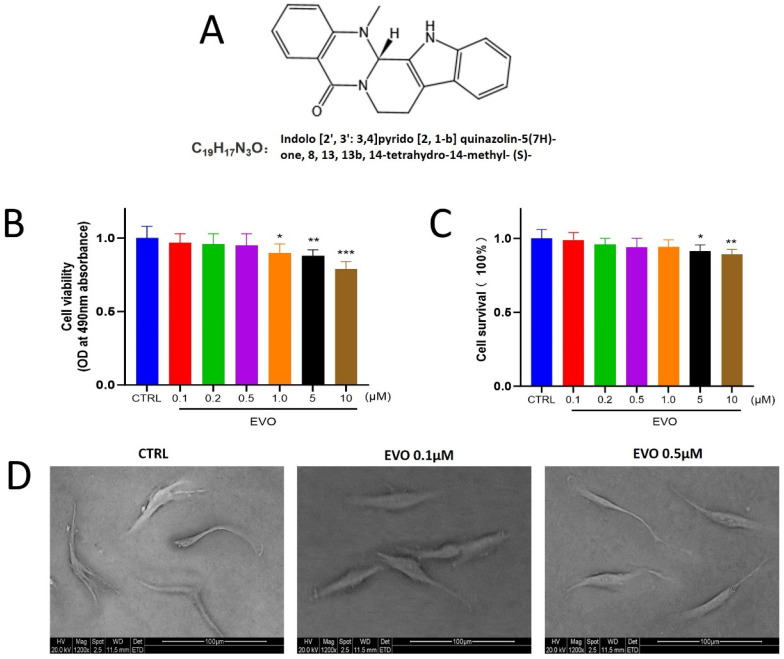
The biocompatibility and cell state of PDLCs after treatment with different concentrations of EVO. (**A**) The molecular formula and formal name (IUPAC) of EVO. (**B**) Analysis of cell viability and survival in PDLCs treated with a gradient of EVO concentrations. (**C**) Assessment of the cytotoxic effects of EVO on PDLCs across a range of concentrations. (**D**) Morphological examination via electron microscopy revealed that PDLCs maintained a typical spindle-shaped fibroblastic appearance in all treatment groups. (*, *p* < 0.05; **, *p* < 0.01; ***, *p* < 0.001).

To determine the cellular response, PDLCs were treated with six concentrations of EVO (Figure 1A) ranging from 0.1 to 10 μM. Results showed no significant differences in cell viability or survival compared to the control group at doses of 0.1 to 0.5 μM and 0.1 to 1.0 μM, respectively (Figure 1B,C). However, both cell viability and survival significantly decreased at higher concentrations, indicating that 0.1 to 0.5 μM EVO represents a suitable experimental range. Scanning electron microscopy (Figure 1D) revealed that PDLCs in all groups exhibited a typical spindle or fusiform morphology with a thickened central region and peripheral pseudopodia extensions. Cell lengths predominantly ranged from 100 to 200 μm, and no obvious morphological differences were observed among groups.

### 3.2. EVO Suppresses IL-1β, TNF-α, IL-6, and NLRP3 Expression

**Figure 2:**
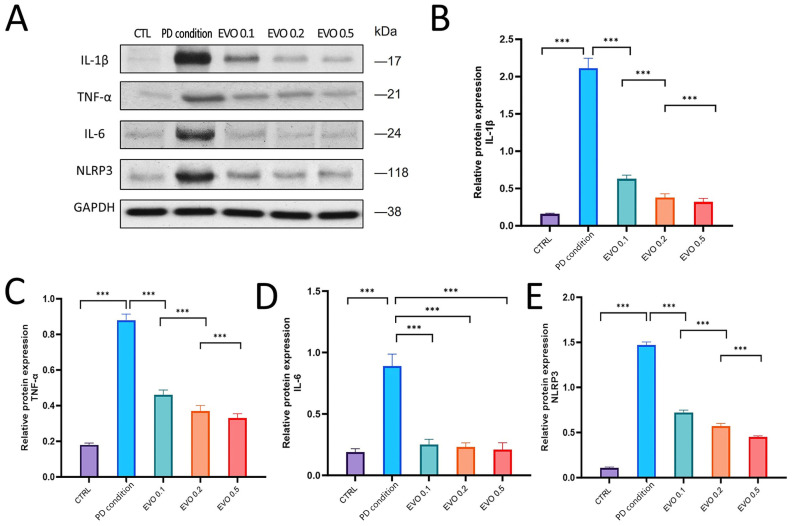
EVO suppresses the expression of IL-1β, TNF-α, IL-6, and NLRP3. (**A**) Western blot analysis showed that PD condition significantly up-regulated the expression of representative cytokines, including IL-1β, TNF-α, IL-6, and NLRP3. (**B**–**E**) Treatment with different concentrations of EVO markedly inhibited this up-regulation in a dose-dependent manner, effectively alleviating the inflammatory response (n = 7). (***, *p* < 0.001).

An inflammatory state in PDLCs was stimulated by LPS treatment. While control PDLCs expressed minimal levels of inflammatory factors, LPS stimulation markedly upregulated the expression of IL-1β, TNF-α, IL-6, and NLRP3 (Figure 2A), by approximately 4- to 10-fold. Treatment with different concentrations of EVO significantly inhibited this upregulation in a dose-dependent manner (Figure 2B–E), effectively alleviating the inflammatory state.

### 3.3. EVO Regulates Oxidative Stress Homeostasis by Suppressing Pro-Oxidative Factors and Promoting Antioxidant Factors

**Figure 3:**
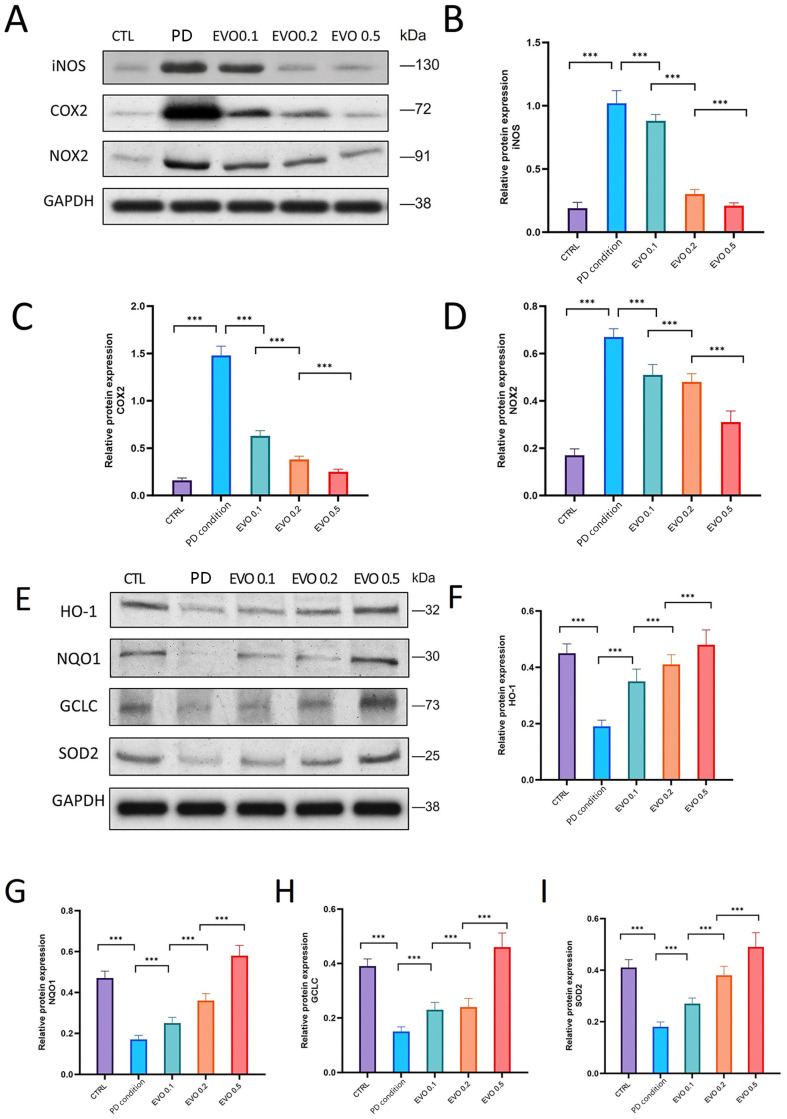
EVO regulates oxidative stress homeostasis by suppressing pro-oxidative factors and promoting antioxidant factors. (**A**) Western blot shows the expression levels of pro-oxidative factors (iNOS, COX2, and NOX2) in the PD condition group and after treatment with different concentrations of EVO. (**B**–**D**) Statistical analysis of the expression levels of iNOS, COX2, and NOX2. (**E**) Western blot shows the expression levels of antioxidant factors (HO-1, NQO1, GCLC, and SOD2) in the PD condition group and after treatment with different concentrations of EVO. (**F**–**I**) Bar graphs showing the statistical values of their expression levels (n = 7). (***, *p* < 0.001).

Under simulated periodontitis conditions, the expression of pro-oxidative factors iNOS, COX2, and NOX2 was markedly increased by approximately 4- to 7-fold (Figure 3A–D), while the levels of antioxidant factors HO-1, NQO1, GCLC, and SOD2 were reduced by about 56% to 63%. EVO treatment significantly upregulated the protein expression of HO-1, NQO1, GCLC, and SOD2 in a dose-dependent manner (Figure 3E–I). Concurrently, it decreased the expression of iNOS, COX2, and NOX2 by approximately 54% to 83%.

### 3.4. EVO Reverses ROS Burst Induced by the Key Periodontal Pathogens

**Figure 4:**
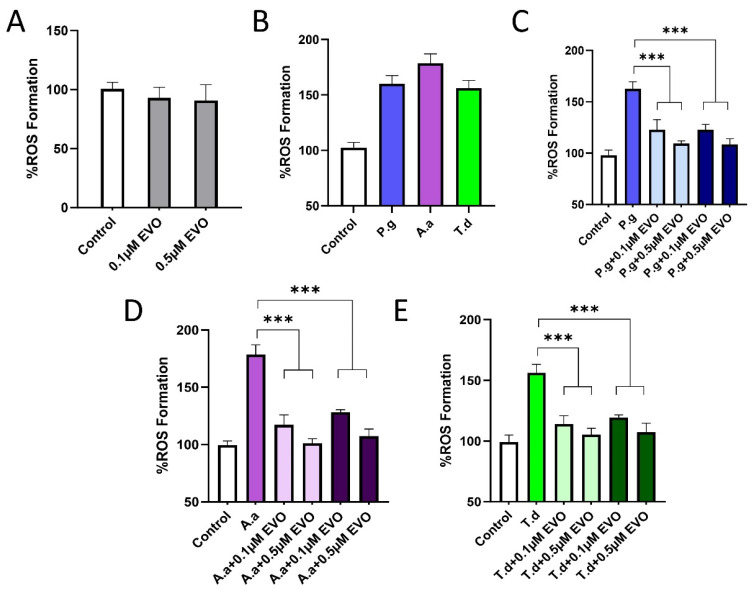
(**A**) ROS expression in healthy PDLCs treated with different concentrations of EVO. (**B**) ROS expression in PDLCs treated with *P. gingivalis*, *A. actinomycetemcomitans*, and *T. denticola*. (**C**) ROS expression in PDLCs incubated with different concentrations of EVO and *P. gingivalis*. Light blue: EVO pretreatment. Dark blue: EVO and *P. gingivalis* co-treatment. (**D**) ROS expression in PDLCs incubated with different concentrations of EVO and *A. actinomycetemcomitans*. Light purple: EVO pretreatment. Dark purple: EVO and *A. actinomycetemcomitans* co-treatment. (**E**) ROS expression in PDLCs incubated with different concentrations of EVO and *T. denticola*. Light green: EVO pretreatment. Dark green: EVO and *T. denticola* co-treatment. n = 7. (***, *p* < 0.001).

The key periodontal pathogens *P. gingivalis*, *A. actinomycetemcomitans*, and *T. denticola* are established major etiological agents of periodontal disease. Treatment of healthy PDLCs with 0.1 or 0.5 μM EVO slightly reduced ROS generation without statistical significance (Figure 4A), indicating no evident oxidative stress toxicity under physiological conditions. On the contrary, infection with each pathogen caused a strong ROS burst (Figure 4B), increasing ROS levels ≥ 1.5-fold. To investigate the optimal timing of EVO administration, both pretreatment and co-treatment strategies were evaluated. Pretreatment with EVO significantly improved the ability of PDLCs to resist infection and inhibited the pathogen-induced ROS burst (Figure 4C–E). Co-treatment also demonstrated strong antioxidant activity, significantly suppressing the pathogen-induced increase in ROS levels. The protective effects of both administration approaches showed a clear concentration-dependence.

### 3.5. EVO Inhibits Plaque Thickness, Mature Plaque Area, and Bacterial Growth of the Key Periodontal Pathogens

**Figure 5:**
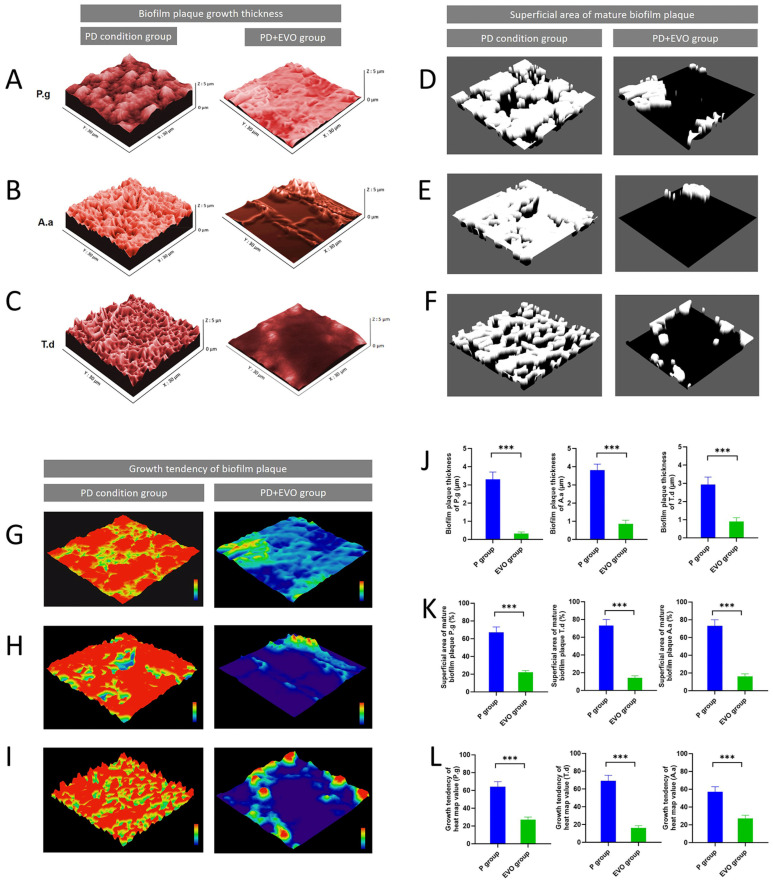
EVO inhibits biofilm thickness, mature plaque area, and bacterial growth trends of periodontal pathogens. (**A**–**C**) The effects of EVO on biofilm thickness were assessed for key periodontal pathogens, *P. gingivalis*, *A. actinomycetemcomitans*, and *T. denticola*. EVO reduced biofilm thickness and increased void spaces. (**D**–**F**) The impact of EVO on mature biofilm zones, which shows a strong inhibition on contiguous mature zones across all three biofilms. (**G**–**I**) Bacterial growth trends were largely inhibited and attenuated by the EVO. (**J**–**L**) Atomic force microscopy results confirmed significantly lower biofilm thickness, mature community area, and attenuated bacterial growth dynamics for all three periodontal pathogens. (n = 7, the concentration of EVO = 0.5 μM, ***, *p* < 0.001).

Under the periodontal condition, the plaque thickness of three periodontal pathogens is significantly elevated (Figure 5A–C). Specifically, the plaque thickness measurements for *P. gingivalis* and *A. actinomycetemcomitans* range between 3 and 4 μm, while that of *T. denticola* approaches 3 μm. EVO treatment significantly decreased the thickness for all three species to below 1 μm. EVO significantly reduced the mature plaque area (Figure 5D–F): coverage of *P. gingivalis* < 30% (67% reduction), *A. actinomycetemcomitans* < 19% (81% reduction), and *T. denticola* < 20% (78% reduction). Furthermore, EVO suppressed the growth trends of the bacteria (Figure 5G–I). The inhibition rates were 58%, 77%, and 53%, respectively. The outcomes depicted in Figure 5J–L reveal that EVO can inhibit the growth of the periodontal pathogens.

### 3.6. Molecular Docking of EVO with KEAP1

**Figure 6:**
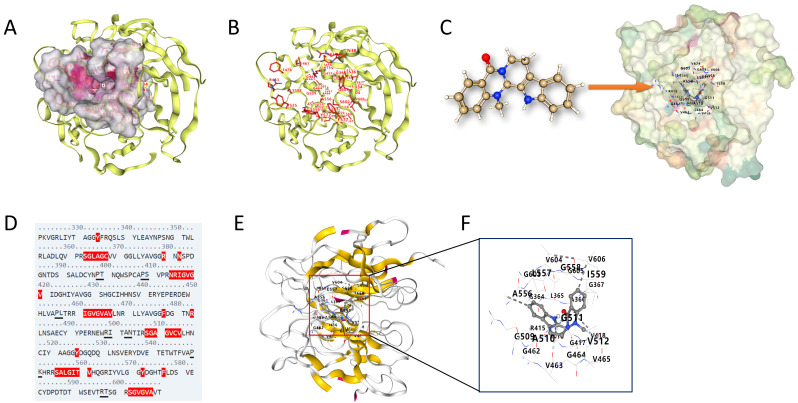
Molecular docking of EVO with KEAP1. (**A**) Molecular architecture of the KEAP1 functional domain and its binding pocket. (**B**) Predicted binding poses of EVO within the functional pocket. (**C**) Molecular interaction between EVO and the KEAP1 protein. (**D**) Sequence motif of the EVO–KEAP1 binding interface. (**E**,**F**) Detailed view of the binding site, highlighting key amino acid residues and their structural interactions.

To further investigate the mechanism of action of EVO, molecular docking was performed between EVO and the DGR domain of KEAP1. The DGR domain forms a pocket-like structure (Figure 6A), with 28 active sites identified within its inner wall energy regions (Figure 6B). The docking analysis (Figure 6C) revealed that EVO binds to the DGR domain of KEAP1 within a binding pocket of 1275 Å^3^, with the interaction center located at coordinates (5, 28, 71) and the pocket dimensions at (19, 18, 21). A total of 17 amino acid residues were involved in direct contact with EVO upon binding (Figure 6D–F).

### 3.7. Validation of the Binding Between EVO and KEAP1

**Figure 7:**
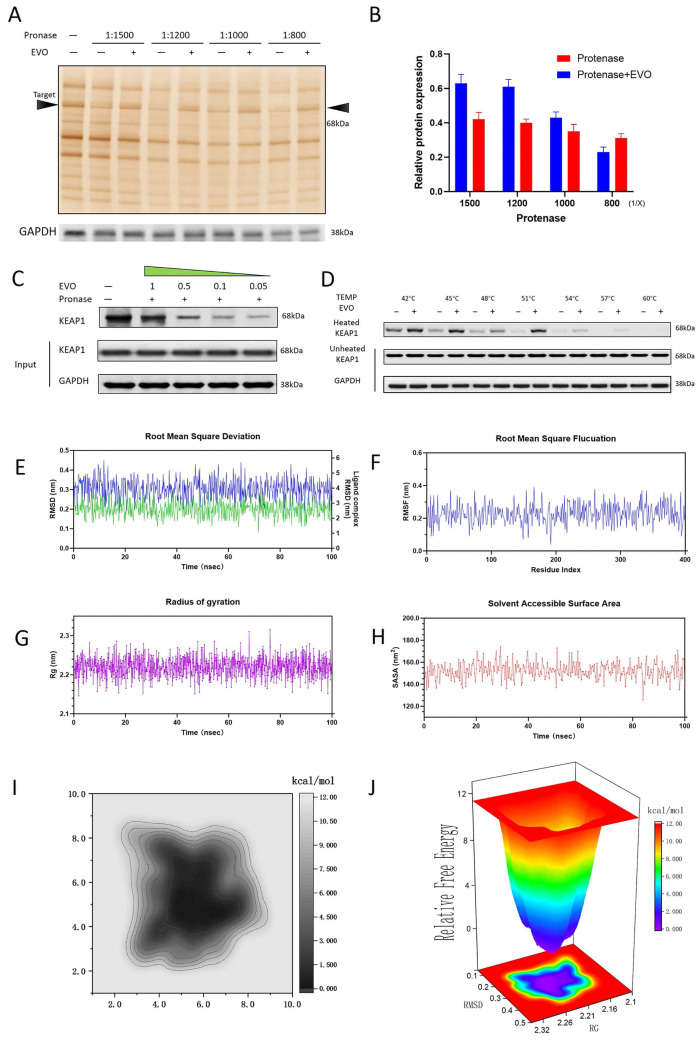
DARTS and flexible molecular docking results. (**A**) DARTS assay identifying KEAP1 as a potential target of EVO (n = 8, the concentration of EVO = 0.5 μM). (**B**) Histogram of data analysis from silver staining results. (**C**) Proteolysis-based validation experiment for the binding affinity of EVO to KEAP1 at different concentrations. Protease concentration = 1:1000. (**D**) Validation experiment for the binding strength of EVO to KEAP1 at different temperatures from 42 to 60 °C. (The concentration of EVO = 0.5 μM). Flexible molecular docking results of the EVO-KEAP1 complex over 100 ns: (**E**) RMSD, (**F**) RMSF, (**G**) Rg, (**H**) SASA. (**I**) Energy distribution diagram of the contact region. (**J**) Free energy landscape projected onto RMSD and radius of gyration under the presence of EVO, showing global minima (ΔG, kJ/mol).

The interaction between EVO and the KEAP1 protein was confirmed using the DARTS assay. Following treatment with a broad concentration range of protease (1:1500 to 1:800), a significant stabilization of KEAP1 against proteolytic degradation was observed in the presence of EVO compared to the solvent control group (Figure 7A,B), as evidenced by the preserved intensity of the protein bands. This protection from proteolysis provides compelling biochemical evidence for a stable binding event between EVO and KEAP1. The protease digestion assay (Figure 7C) showed that the binding of EVO to KEAP1 can significantly protect against proteolytic hydrolysis. The validation experiment for the binding strength of EVO to KEAP1 at different temperatures (Figure 7D) showed that as the temperature increased from 42 °C to 60 °C, KEAP1 was progressively degraded. However, the binding of EVO to KEAP1 significantly inhibited the temperature-induced protein degradation.

### 3.8. Molecular Dynamics Simulations and Free Energy Calculations of the EVO-KEAP1 Complex

To clarify the interaction mechanism, 100 ns all-atom molecular dynamics simulations were performed. The system quickly achieved equilibrium and remained stable during the simulation trajectory, as indicated by a stable protein backbone RMSD of 0.211 nm (Figure 7E) and an RMSF of 0.226 nm (Figure 7F). The corresponding ligand RMSD for EVO was 0.294 nm, indicating a stable binding pose within the KEAP1 pocket. Further confirming structural integrity, the Rg remained steady at 0.218 nm (Figure 7G). Consistent with these findings, SASA analysis (Figure 7H) showed a stable average value of 152.3 nm^2^. Thermodynamic analysis (Figure 7I,J) revealed a single, deep, and well-defined global energy minimum in the free energy landscape (FEL), indicating that the EVO-KEAP1 complex predominantly samples a singular low-energy and highly stable conformational state.

### 3.9. Apoptosis Analysis by Flow Cytometry

**Figure 8:**
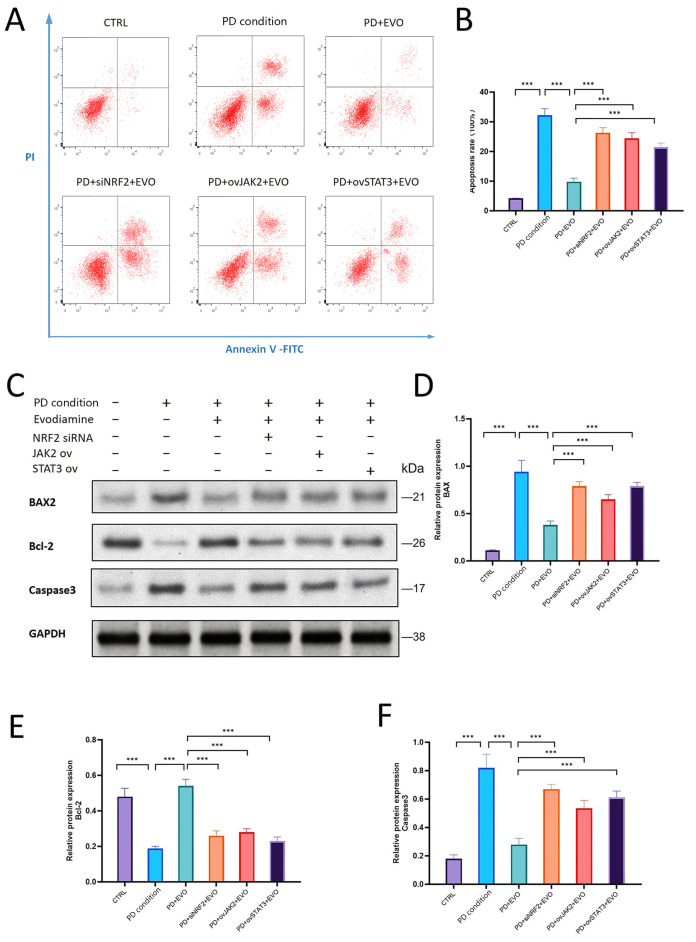
EVO regulates apoptosis by activating NRF2 and inhibiting JAK2/STAT3 pathways. (**A**,**B**) Flow cytometric detection of apoptosis demonstrated the anti-apoptotic effect of EVO. The periodontitis condition group showed a significant increase in apoptosis, which was reduced by EVO treatment. NRF2 knockdown or JAK2/STAT3 overexpression abolished this protection. (**C**) Western blot analysis was performed on key apoptosis regulatory proteins. (**D**–**F**) The periodontitis condition group showed increased BAX and cleaved caspase-3 and decreased Bcl-2. EVO reversed these changes. NRF2 silencing or JAK2/STAT3 overexpression blocked EVO’s effects. n = 7; the concentration of EVO = 0.5 μM. (***, *p* < 0.001).

Consistent with the protein expression findings, flow cytometric analysis of apoptosis (Figure 8A,B) yielded corroborative results. The periodontitis group exhibited a pronounced increase in total apoptosis, with significant accumulation of cells in both early and late apoptotic quadrants. EVO treatment decreased the percentage of apoptotic cells. However, NRF2 knockdown almost completely abrogated this protective effect, resulting in a significant increase in apoptotic cells. Furthermore, overexpression of either JAK2 or STAT3 blocked the protective effect of EVO, leading to increased apoptosis of PDLCs.

### 3.10. EVO Regulates Apoptosis by Activating NRF2 and Inhibiting JAK2/STAT3 Pathways

Western blot analysis evaluated the expression of BAX, Bcl-2, and cleaved caspase-3 (Figure 8C). The periodontitis group showed a significant pro-apoptotic phenotype, with an increase of BAX and cleaved caspase-3 involved and a decrease of Bcl-2. These changes were reversed by EVO treatment, giving a pro-survival balance. NRF2 silencing blocked the protective effects of EVO, resulting in a re-elevation of BAX and cleaved caspase-3 and a reduction of Bcl-2 compared to the EVO group. Furthermore, overexpression of JAK2 or STAT3 abolished the anti-apoptotic effect of EVO (Figure 8D–F), leading to increased BAX and cleaved caspase-3 and a decrease in Bcl-2.

### 3.11. EVO Alleviates G0/G1 Phase Cell Cycle Arrest via NRF2 and JAK2 Pathways

**Figure 9:**
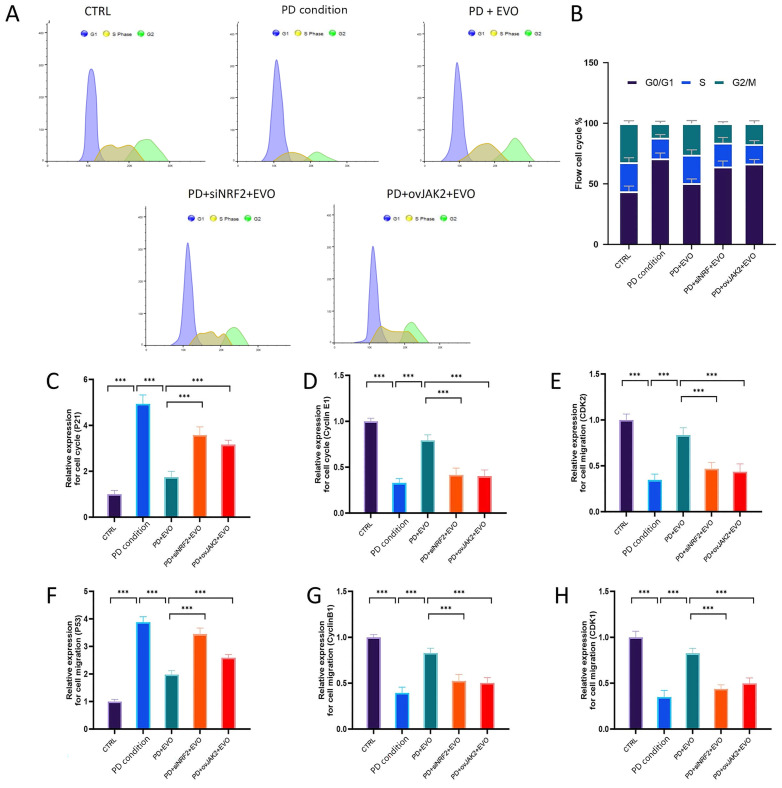
Cell cycle analysis and expression of cell cycle-related factors. (**A**,**B**) Flow cytometry showed increased G0/G1 phase arrest in the PD condition group (27.2%). EVO treatment alleviated this arrest, decreasing G0/G1 phase cells by 28.7%, and increasing S and G2/M phases cells. (**C**–**H**) qRT-PCR results showed decreased *Cyclin E1* and *CDK2* and increased *p21* and *p53* in the PD condition group. EVO upregulated *Cyclin B1* and *CDK1*. Knockdown of NRF2 abolished EVO’s effects, while overexpression of either JAK2 or STAT3 restored G0/G1 arrest. n = 7; the concentration of EVO = 0.5 μM. (***, *p* < 0.001).

Flow cytometry analysis revealed that the periodontitis group manifested cell cycle arrest in the G0/G1 phase, which was a significant increase by 27.2% compared to the control (Figure 9A,B). This observation was confirmed by real-time PCR data (Figure 9C–H), which illustrated the reduced expression of *Cyclin E1* and *CDK2* and enhanced levels of *p21* and *p53*. EVO treatment significantly reduced the cell cycle arrest by decreasing the G0/G1 phase proportions by 28.7% and increasing the S and G2/M phase proportions. In addition, the expression of *Cyclin B1* and *CDK1* was upregulated by EVO. Nevertheless, siRNA knockdown of NRF2 negated the protective effects of EVO on cell cycle, while JAK2 overexpression restored G0/G1 phase arrest. These results suggest that the KEAP1/NRF2 couple regulates cell cycle arrest through the JAK2/STAT3 signaling pathway.

### 3.12. EVO Regulates PDLC Migration and Cytoskeleton via Activating NRF2 and Inhibiting JAK2

**Figure 10:**
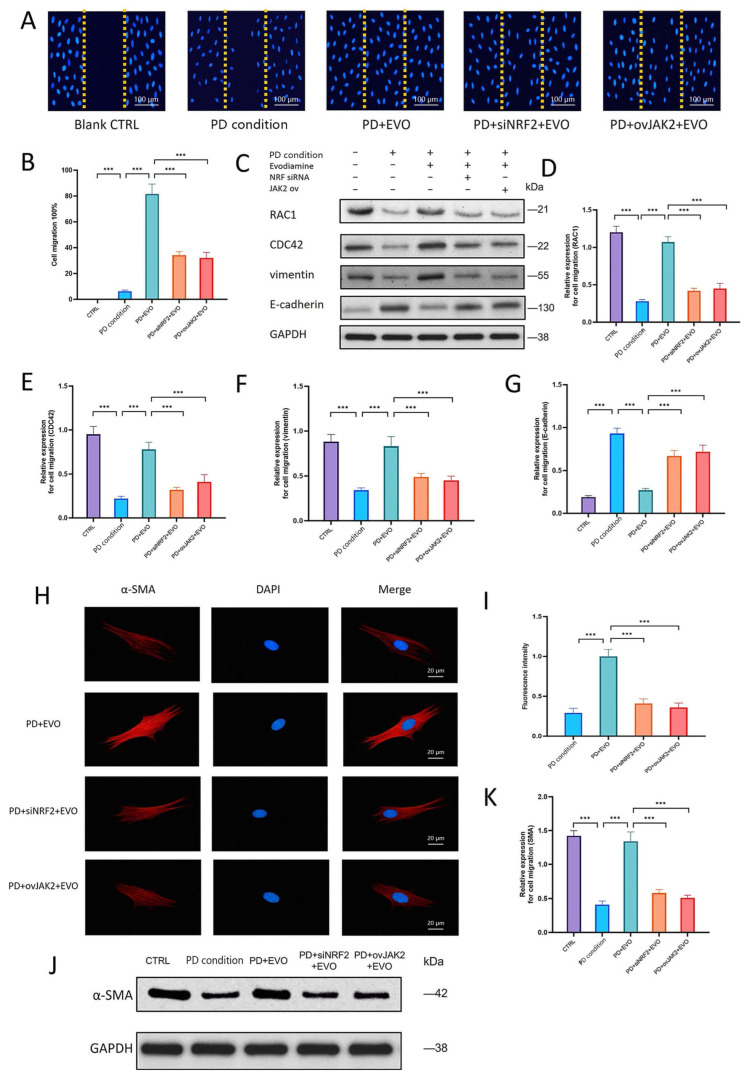
EVO regulates PDLC cell migration and cytoskeleton by activating NRF2 and inhibiting JAK2. (**A**,**B**) Scratch assay results. EVO increased migration by 92% compared to the PD condition group. Migration was lower in NRF2-knockdown and JAK2-overexpression groups. (**C**–**G**) Western blot analysis of migration proteins. EVO increased RAC1 and CDC42 expression and reversed vimentin upregulation and E-cadherin downregulation. These effects were reversed in NRF2-knockdown and JAK2-overexpression groups. (**H**,**I**) Immunofluorescence staining for α-SMA. EVO-treated cells showed prominent α-SMA stress fibers, which were weaker in NRF2-knockdown and JAK2-overexpression groups. (**J**,**K**) Western blot results for α-SMA demonstrated the same trend of changes. (***, *p* < 0.001).

Wound healing assays demonstrated that EVO enhanced PDLC migration by 92% compared to the PD group (Figure 10A,B). This effect was significantly attenuated in NRF2-knockdown and JAK2-overexpression groups, which showed increases of only 52% and 61%, respectively. Western blot analysis (Figure 10C–G) further revealed that EVO upregulated the migration-related proteins RAC1 and CDC42 and reversed the PD-induced expression changes in vimentin and E-cadherin. These regulatory effects were abolished upon NRF2 knockdown or JAK2 overexpression. Immunofluorescence staining showed that EVO robustly promoted α-SMA stress fiber assembly, increasing its mean fluorescence intensity by approximately 59% (Figure 10H–K). This enhancement was similarly reversed by NRF2 knockdown or JAK2 overexpression, a trend confirmed by Western blot. Collectively, the results indicate that EVO regulates PDLC migration and cytoskeletal integrity through activating NRF2 and inhibiting JAK2.

## 4. Discussion

Periodontitis is an irreversible inflammatory disease that ultimately leads to alveolar bone resorption, tooth loosening, and loss, affecting approximately 62% of the global population [24]. Oxidative stress plays a critical role in its pathogenesis [25,26]. This study provides molecular dynamics and biochemical evidence of the binding of EVO to KEAP1. By disrupting the KEAP1–NRF2 interaction, EVO enhances the cellular antioxidant defense system to eliminate excess reactive oxygen species (ROS). Furthermore, EVO modulates inflammation in PDLCs through synergistic multi-pathway mechanisms.

EVO did not exhibit cytotoxicity to healthy PDLCs across a wide concentration range. Three-dimensional AFM analysis of its antibacterial properties revealed that EVO inhibited the three major periodontal pathogens. Mature biofilms dissociated from a dense, continuous state into dispersed microcolonies. Corroborating data confirmed EVO’s efficacy in inhibiting pathogen spread. According to Berto et al., a 40–50% reduction in plaque thickness substantially disrupts the bacterial community, weakens synergistic interactions, and protects periodontal ligament cells [27,28].

In terms of oxidative stress regulation, our results showed that EVO effectively reversed the elevated ROS levels induced by three major periodontal pathogens—*P. gingivalis*, *A. actinomycetemcomitans*, and *T. denticola*—under both pretreatment and cotreatment conditions. EVO significantly suppressed the expression of iNOS, COX2, and NOX2 in the inflammatory microenvironment. Downregulation of iNOS reduced peroxynitrite production, while inhibition of COX2 decreased prostaglandin generation [29,30,31]. As NOX2 serves as a major source of reactive oxygen species, suppression of its expression directly decreased superoxide anion generation [32], providing a possible molecular explanation for the decline in ROS levels. Concurrently, EVO upregulated downstream antioxidant enzymes, including HO-1, NQO1, GCLC, and SOD2, indicating enhanced cellular defense via the NRF2-mediated antioxidant network [33,34]. Moreover, EVO markedly downregulate the expression levels of pro-inflammatory cytokines IL-1β, IL-6, and TNF-α, demonstrating a synergistic anti-inflammatory and antioxidant effect.

KEAP1 serves as an adapter protein in the Cullin3-RING E3 ubiquitin ligase complex that recognizes NRF2 and promotes its ubiquitination and degradation [35]. The NRF2-binding interface is formed by the BTB domain and the six-bladed β-propeller structure of the Kelch domain [36]. The ETGE and DLG motifs of NRF2 interact with this interface, leading to ubiquitination and subsequent inhibition and degradation of NRF2 [17,35]. Our findings indicate that the EVO molecule precisely binds into a hydrophobic pocket of KEAP1, thereby disrupting its normal interaction with NRF2. Consequently, NRF2 is released and stabilized due to impaired KEAP1-mediated ubiquitination, leading to its activation.

Molecular dynamics simulations confirmed the conformational stability of the EVO-KEAP1 complex, with an RMSD maintained below 0.3 nm. Low residue fluctuation at the binding interface indicates the formation of a stable protein–ligand complex. A dissociation constant (Kd) < 1 μM corresponds to a binding energy < −8 kcal/mol, indicating stable molecular binding, while a binding free energy < −10 kcal/mol signifies strong binding [37]. Our results show that the binding energy of EVO-KEAP1 reaches −11.67 kcal/mol, approaching the threshold for very strong binding (−12 kcal/mol). Furthermore, the free energy landscape exhibits a single, deep, and well-defined global energy minimum. The activation of the antioxidant defense system triggered by this binding may represent the initial molecular event through which EVO subsequently exerts protective effects such as anti-inflammatory and anti-apoptotic actions.

As pivotal regulators of oxidative stress homeostasis, KEAP1/NRF2 can exert further biological regulation through certain immune or inflammatory signaling pathways, including the JAK2/STAT3 pathway [38,39]. The JAK2/STAT3 pathway, involved in diverse cellular processes, serves as a critical regulator of apoptosis, a “double-edged sword” in both physiological and pathological contexts [40,41]. Apoptosis is important for the maintenance of tissue homeostasis under normal conditions. In chronic inflammatory diseases such as periodontitis, excessive apoptosis of PDLCs contributes to tissue destruction and loss of structural integrity [42]. According to our findings, JAK2/STAT3 pathway acts as a pro-apoptotic factor, and EVO alleviates PDLC apoptosis. The observed reduction in the protective effect of EVO following JAK2/STAT3 overexpression clearly confirms the role of this pathway in driving PDLC death in this environment. This regulation of apoptosis stands in sharp contrast to its previously demonstrated oncogenic role in certain cancers [43]. Our results further demonstrate that EVO significantly upregulates Bcl-2 expression while suppressing BAX activation and caspase-3 cleavage, indicating that EVO promotes PDLCs survival via modulation of the JAK2/STAT3 pathway. JAK2 upregulates the expression of the anti-apoptotic protein Bcl-2 through the transcription factor STAT3 [44,45]. Bcl-2, primarily localized on the outer mitochondrial membrane, directly antagonizes BAX to prevent mitochondrial pore formation, thereby stabilizing mitochondrial membrane integrity [46]. This further inhibits the function of the downstream executioner caspase-3, preventing the degradation of cellular substrates, alleviating DNA fragmentation and cellular disintegration, and ultimately promoting PDLCs survival [45,46].

Our results showed that under periodontitis conditions, cell cycle arrest at the G0/G1 phase increased by 27.2%. This arrest prevents cells from further maturation and division [47,48], thereby hindering the recovery of PDLCs. EVO significantly upregulated the expression of key regulators for G1/S transition (Cyclin E1 and CDK2) and G2/M transition (Cyclin B1 and CDK1), while downregulating the levels of cycle inhibitors p53 and p21. Consequently, the proportion of cells in the G0/G1 phase decreased by 28.7%, with an increase in the number of cells in the S and G2/M phases. On one hand, EVO may promote cyclin expression and inhibit the p53-p21 axis by modulating the JAK2/STAT3 pathway [47,48,49]. On the other hand, as redox homeostasis is also a critical factor influencing the cell cycle [50], EVO likely helps maintain intracellular redox balance through the KEAP1/NRF2 pathway, thereby creating a favorable condition for cell cycle progression.

The scratch assay results showed that EVO enhanced the directional migration of PDLCs, possibly through the NRF2 and JAK2/STAT3 pathways, which are involved in cytoskeletal reorganization [51] and pseudopodial migration [49]. Western blot analysis supported this observation, demonstrating that EVO strongly upregulated the key Rho GTPases RAC1 and CDC42, which regulate cytoskeletal reorganization [52] and pseudopod formation [53,54], respectively. Regarding cell phenotypic markers, EVO treatment reversed the periodontitis-induced upregulation of vimentin and downregulation of E-cadherin. Together, these data suggest a dual mechanism: EVO promotes cell motility via the JAK2/STAT3–RAC1/CDC42 axis, while also maintaining intercellular junctions and stabilizing cell phenotype by activating the KEAP1/NRF2 pathway.

This study has limitations. Firstly, the in vitro model using PDLCs partially simulates the state of periodontitis and cannot fully replicate the multifactorial in vivo microenvironment. Thus, further evaluation is required to fully understand the role of EVO. Secondly, donor-related variables such as age in the primary cell sources may influence the antioxidant responses observed in isolated PDLCs. Thirdly, the concentration of EVO that exhibits pharmacological activity in cell culture may not necessarily translate to therapeutically effective concentrations in vivo. The optimal treatment concentration of EVO warrants further investigation.

## 5. Conclusions

In conclusion, EVO promotes NRF2 expression and modulates redox homeostasis by binding to the Kelch domain of KEAP1 with a strong binding energy of −11.67 kcal/mol. It modulates proinflammatory cytokine expression, apoptosis, cell cycle distribution, cytoskeletal dynamics, and cell migration via the JAK2/STAT3 signaling pathway. Furthermore, EVO significantly inhibits the thickness, coverage, and growth of key periodontal pathogens biofilms, while scavenging ROS to enhance antioxidant defense. These findings advance our understanding of the antioxidant mechanisms of EVO.

## Data Availability

The original contributions presented in this study are included in the article/Supplementary Materials. Further inquiries can be directed to the corresponding author.

## Supplementary Materials

The following supporting information can be downloaded at https://www.mdpi.com/article/10.3390/antiox15040471/s1: Table S1: Detailed concentrations and sources of antibodies in Western blot Analysis; Table S2: Characteristics of primers used in RT-qPCR.

## References

[1] Peres M.A., Macpherson L.M.D., Weyant R.J., Daly B., Venturelli R., Mathur M.R., Listl S., Celeste R.K., Guarnizo-Herreno C.C., Kearns C. (2019). Oral diseases: A global public health challenge. Lancet.

[2] Könönen E., Gursoy M., Gursoy U.K. (2019). Periodontitis: A Multifaceted Disease of Tooth-Supporting Tissues. J. Clin. Med..

[3] Sczepanik F.S.C., Grossi M.L., Casati M., Goldberg M., Glogauer M., Fine N., Tenenbaum H.C. (2020). Periodontitis is an inflammatory disease of oxidative stress: We should treat it that way. Periodontol. 2000.

[4] Usui M., Onizuka S., Sato T., Kokabu S., Ariyoshi W., Nakashima K. (2021). Mechanism of alveolar bone destruction in periodontitis-Periodontal bacteria and inflammation. Jpn. Dent. Sci. Rev..

[5] Sena C.M., Leandro A., Azul L., Seiça R., Perry G. (2018). Vascular Oxidative Stress: Impact and Therapeutic Approaches. Front. Physiol..

[6] Gomez-Mejiba S.E., Zhai Z., Akram H., Pye Q.N., Hensley K., Kurien B.T., Scofield R.H., Ramirez D.C. (2009). Inhalation of environmental stressors & chronic inflammation: Autoimmunity and neurodegeneration. Mutat. Res..

[7] Hong Y., Boiti A., Vallone D., Foulkes N.S. (2024). Reactive Oxygen Species Signaling and Oxidative Stress: Transcriptional Regulation and Evolution. Antioxidants.

[8] Finkel T., Holbrook N.J. (2000). Oxidants, oxidative stress and the biology of ageing. Nature.

[9] Salminen A., Ojala J., Kaarniranta K., Kauppinen A. (2012). Mitochondrial dysfunction and oxidative stress activate inflammasomes: Impact on the aging process and age-related diseases. Cell. Mol. Life Sci. CMLS.

[10] Li X., Ge J., Zheng Q., Zhang J., Sun R., Liu R. (2020). Evodiamine and rutaecarpine from Tetradium ruticarpum in the treatment of liver diseases. Phytomedicine Int. J. Phytother. Phytopharm..

[11] Luo C., Ai J., Ren E., Li J., Feng C., Li X., Luo X. (2021). Research progress on evodiamine, a bioactive alkaloid of Evodiae fructus: Focus on its anti-cancer activity and bioavailability (Review). Exp. Ther. Med..

[12] Fan X., Zhu J.Y., Sun Y., Luo L., Yan J., Yang X., Yu J., Tang W.Q., Ma W., Liang H.P. (2017). Evodiamine Inhibits Zymosan-Induced Inflammation In Vitro and In Vivo: Inactivation of NF-kappaB by Inhibiting IkappaBalpha Phosphorylation. Inflammation.

[13] Kuai J., Zhang N. (2022). Upregulation of SIRT1 by Evodiamine activates PI3K/AKT pathway and blocks intervertebral disc degeneration. Mol. Med. Rep..

[14] Li C.G., Zeng Q.Z., Chen M.Y., Xu L.H., Zhang C.C., Mai F.Y., Zeng C.Y., He X.H., Ouyang D.Y. (2019). Evodiamine Augments NLRP3 Inflammasome Activation and Anti-bacterial Responses Through Inducing alpha-Tubulin Acetylation. Front. Pharmacol..

[15] Wu P., Chen Y. (2019). Evodiamine ameliorates paclitaxel-induced neuropathic pain by inhibiting inflammation and maintaining mitochondrial anti-oxidant functions. Hum. Cell.

[16] Zhang D.D., Lo S.C., Cross J.V., Templeton D.J., Hannink M. (2004). Keap1 is a redox-regulated substrate adaptor protein for a Cul3-dependent ubiquitin ligase complex. Mol. Cell. Biol..

[17] Canning P., Sorrell F.J., Bullock A.N. (2015). Structural basis of Keap1 interactions with Nrf2. Free Radic. Biol. Med..

[18] Itoh K., Mimura J., Yamamoto M. (2010). Discovery of the negative regulator of Nrf2, Keap1: A historical overview. Antioxid. Redox Signal..

[19] Kobayashi A., Kang M.I., Okawa H., Ohtsuji M., Zenke Y., Chiba T., Igarashi K., Yamamoto M. (2004). Oxidative stress sensor Keap1 functions as an adaptor for Cul3-based E3 ligase to regulate proteasomal degradation of Nrf2. Mol. Cell. Biol..

[20] Ni J., Yang D., Song L., Li C. (2016). Protective effects of paeoniflorin on alveolar bone resorption and soft-tissue breakdown in experimental periodontitis. J. Periodontal Res..

[21] Mohammad C.A., Ali K.M., Sha A.M., Gul S.S. (2022). Antioxidant Effects of Curcumin Gel in Experimental Induced Diabetes and Periodontitis in Rats. BioMed Res. Int..

[22] Shin J.W., Chun K.S., Kim D.H., Kim S.J., Kim S.H., Cho N.C., Na H.K., Surh Y.J. (2020). Curcumin induces stabilization of Nrf2 protein through Keap1 cysteine modification. Biochem. Pharmacol..

[23] Cheng Y., Cheng L., Gao X., Chen S., Wu P., Wang C., Liu Z. (2021). Covalent modification of Keap1 at Cys77 and Cys434 by pubescenoside a suppresses oxidative stress-induced NLRP3 inflammasome activation in myocardial ischemia-reperfusion injury. Theranostics.

[24] Kwon T., Lamster I.B., Levin L. (2021). Current Concepts in the Management of Periodontitis. Int. Dent. J..

[25] Tóthová L., Celec P. (2017). Oxidative Stress and Antioxidants in the Diagnosis and Therapy of Periodontitis. Front. Physiol..

[26] Sies H. (2015). Oxidative stress: A concept in redox biology and medicine. Redox Biol..

[27] Berto L.A., Ettmayer J.B., Stutzer D., Nietzsche S., Niederhauser T., Burger J., Sculean A., Eick S., Hofmann M. (2024). In-vitro effects of novel periodontal scalers with a planar ultrasonic piezoelectric transducer on periodontal biofilm removal, dentine surface roughness, and periodontal ligament fibroblasts adhesion. Clin. Oral Investig..

[28] Socransky S.S., Haffajee A.D., Cugini M.A., Smith C., Kent R.L. (1998). Microbial complexes in subgingival plaque. J. Clin. Periodontol..

[29] Gallorini M., Rapino M., Schweikl H., Cataldi A., Amoroso R., Maccallini C. (2021). Selective Inhibitors of the Inducible Nitric Oxide Synthase as Modulators of Cell Responses in LPS-Stimulated Human Monocytes. Molecules.

[30] Kawano T., Kunz A., Abe T., Girouard H., Anrather J., Zhou P., Iadecola C. (2007). iNOS-derived NO and nox2-derived superoxide confer tolerance to excitotoxic brain injury through peroxynitrite. J. Cereb. Blood Flow Metab. Off. J. Int. Soc. Cereb. Blood Flow Metab..

[31] Li Y., Soendergaard C., Bergenheim F.H., Aronoff D.M., Milne G., Riis L.B., Seidelin J.B., Jensen K.B., Nielsen O.H. (2018). COX-2-PGE(2) Signaling Impairs Intestinal Epithelial Regeneration and Associates with TNF Inhibitor Responsiveness in Ulcerative Colitis. EBioMedicine.

[32] Winterbourn C.C., Kettle A.J., Hampton M.B. (2016). Reactive Oxygen Species and Neutrophil Function. Annu. Rev. Biochem..

[33] Loboda A., Damulewicz M., Pyza E., Jozkowicz A., Dulak J. (2016). Role of Nrf2/HO-1 system in development, oxidative stress response and diseases: An evolutionarily conserved mechanism. Cell. Mol. Life Sci..

[34] Zhang Q.-Y., Chu X.-Y., Jiang L.-H., Liu M.-Y., Mei Z.-L., Zhang H.-Y. (2017). Identification of Non-Electrophilic Nrf2 Activators from Approved Drugs. Molecules.

[35] Bellezza I., Giambanco I., Minelli A., Donato R. (2018). Nrf2-Keap1 signaling in oxidative and reductive stress. Biochim. Biophys. Acta Mol. Cell Res..

[36] Abed D.A., Goldstein M., Albanyan H., Jin H., Hu L. (2015). Discovery of direct inhibitors of Keap1-Nrf2 protein-protein interaction as potential therapeutic and preventive agents. Acta Pharm. Sin. B.

[37] Wells J.A., McClendon C.L. (2007). Reaching for high-hanging fruit in drug discovery at protein-protein interfaces. Nature.

[38] Popov S.V., Mukhomedzyanov A.V., Voronkov N.S., Derkachev I.A., Boshchenko A.A., Fu F., Sufianova G.Z., Khlestkina M.S., Maslov L.N. (2023). Regulation of autophagy of the heart in ischemia and reperfusion. Apoptosis.

[39] El Safadi M., Hayat M.F., Akbar A., Nisar A., Alzahrani F.M., Alzahrani K.J. (2024). Pharmacotherapeutic potential of bilobetin to combat chromium induced hepatotoxicity via regulating TLR-4, Nrf-2/Keap-1, JAK1/STAT3 and NF-kappaB pathway: A pharmacokinetic and molecular dynamic approach. J. Trace Elem. Med. Biol..

[40] Hu X., Li J., Fu M., Zhao X., Wang W. (2021). The JAK/STAT signaling pathway: From bench to clinic. Signal Transduct. Target. Ther..

[41] Mengie Ayele T., Tilahun Muche Z., Behaile Teklemariam A., Bogale Kassie A., Chekol Abebe E. (2022). Role of JAK2/STAT3 Signaling Pathway in the Tumorigenesis, Chemotherapy Resistance, and Treatment of Solid Tumors: A Systemic Review. J. Inflamm. Res..

[42] Shi J., Li J., Su W., Zhao S., Li H., Lei L. (2019). Loss of periodontal ligament fibroblasts by RIPK3-MLKL-mediated necroptosis in the progress of chronic periodontitis. Sci. Rep..

[43] Bournazou E., Bromberg J. (2013). Targeting the tumor microenvironment: JAK-STAT3 signaling. JAK-STAT.

[44] Singh P., Lim B. (2022). Targeting Apoptosis in Cancer. Curr. Oncol. Rep..

[45] Mustafa M., Ahmad R., Tantry I.Q., Ahmad W., Siddiqui S., Alam M., Abbas K., Moinuddin, Hassan M.I., Habib S. (2024). Apoptosis: A Comprehensive Overview of Signaling Pathways, Morphological Changes, and Physiological Significance and Therapeutic Implications. Cells.

[46] Wang B., Wang Y., Zhang J., Hu C., Jiang J., Li Y., Peng Z. (2023). ROS-induced lipid peroxidation modulates cell death outcome: Mechanisms behind apoptosis, autophagy, and ferroptosis. Arch. Toxicol..

[47] Zhang N., Zheng Q., Wang Y., Lin J., Wang H., Liu R., Yan M., Chen X., Yang J., Chen X. (2021). Renoprotective Effect of the Recombinant Anti-IL-6R Fusion Proteins by Inhibiting JAK2/STAT3 Signaling Pathway in Diabetic Nephropathy. Front. Pharmacol..

[48] Romeo M.A., Gilardini Montani M.S., Benedetti R., Santarelli R., D’Orazi G., Cirone M. (2020). STAT3 and mutp53 Engage a Positive Feedback Loop Involving HSP90 and the Mevalonate Pathway. Front. Oncol..

[49] Yu H., Pardoll D., Jove R. (2009). STATs in cancer inflammation and immunity: A leading role for STAT3. Nat. Rev. Cancer.

[50] Conour J.E., Graham W.V., Gaskins H.R. (2004). A combined in vitro/bioinformatic investigation of redox regulatory mechanisms governing cell cycle progression. Physiol. Genom..

[51] Gao H., Tang P., Ni K., Zhu L., Chen S., Zheng Y., Wan Y. (2021). Inhibition of Kelch-like epichlorohydrin-related protein 1 promotes the progression and drug resistance of lung adenocarcinoma. PeerJ.

[52] Polimeni G., Xiropaidis A.V., Wikesjö U.M. (2006). Biology and principles of periodontal wound healing/regeneration. Periodontol. 2000.

[53] Corry J., Mott H.R., Owen D. (2020). Activation of STAT transcription factors by the Rho-family GTPases. Biochem. Soc. Trans..

[54] Cox D., Chang P., Zhang Q., Reddy P.G., Bokoch G.M., Greenberg S. (1997). Requirements for both Rac1 and Cdc42 in membrane ruffling and phagocytosis in leukocytes. J. Exp. Med..

