# Supplementary material for: Evodiamine Regulates Oxidative Stress and the JAK2/STAT3 Pathway to Modulate Apoptosis, Inflammation, Cell Cycle Arrest, and Migration in Periodontal Ligament Cells
Source: Antioxidants (Basel). 2026 Apr 10;15(4):471. doi: 10.3390/antiox15040471 (PMC13113711; doi:10.3390/antiox15040471)
Supplement: Supplementary file 1 [file antioxidants-15-00471-s001.zip › Supplementary Table S1.pdf]

Supplementary Table S1

Detailed concentrations and sources of antibodies in western blot analysis

Target Protein	Dilution	Source/Clone
**BAX2**	1:2000	Abcam (ab32503)
**Bcl-2**	1:1000	Abcam (ab32124)
**Caspase-3**	1:2000	Abcam (ab32042)
**RAC1**	1:1000	CST (#2465)
**CDC42**	1:1000	CST (#2466)
**Vimentin**	1:500	Abcam (ab92547)
**E-cadherin**	1:1000	CST (#3195)
**α-SMA**	1:500	R&D Systems (MAB5655)
**IL-1β**	1:1000	Abcam (ab216995)
**TNF-α**	1:1000	Abcam (ab183218)
**IL-6**	1:1000	Abcam (ab9423)
**iNOS**	1:2000	CST (#13118)
**COX-2**	1:2000	CST (#160108)
**NOX2**	1:1000	CST (#3868)
**HO-1**	1:500	Abcam (ab13243)

**NQO1**	1:500	Abcam (ab2346)
**GCLC**	1:1000	Abcam (ab53179)
**SOD2**	1:1000	CST (#13141)
**GAPDH**	1:5000	CST (#2118)
