# Supplementary material for: Evodiamine Regulates Oxidative Stress and the JAK2/STAT3 Pathway to Modulate Apoptosis, Inflammation, Cell Cycle Arrest, and Migration in Periodontal Ligament Cells
Source: Antioxidants (Basel). 2026 Apr 10;15(4):471. doi: 10.3390/antiox15040471 (PMC13113711; doi:10.3390/antiox15040471)
Supplement: Supplementary file 1 [file antioxidants-15-00471-s001.zip › Supplementary Table S2.pdf]

Supplementary Table S2

Characteristics of primers used in RT-qPCR

Gene symbol	Sequence (5'→3')	
GAPDH	Sense	GCCATGTACGTAGCCATCCA
	Antisense	GAACCGCTCATTGCCGATAG
P21	Sense	TGTCCGTCAGAACCCATGC
	Antisense	AAAGTCGAAGTTCCATCGCTC
P53	Sense	CCTCAGCATCTTATCCGAGTGG
	Antisense	TGGATGGTGTGTGTTACAGCAGC
Cyclin E1	Sense	TGTGTCCTGGATGTTGACTGCC
	Antisense	CTCTATGTCGCACCACTGATACC
Cyclin B1	Sense	AACTTTCGCCTGAGCCTATTTT
	Antisense	TTGGTCTGACTGCTTGCTCTT
CDK1	Sense	GGAAACCAGGAAGCCTAGCATC
	Antisense	GGATGATTCAGTGCCATTTTTTGCC
CDK2	Sense	ATGGATGCCTCTGCTCTCACTG
	Antisense	CCCGATGAGAATGGCAGAAAGC
